# Meet the authors: Daisuke Komura and Shumpei Ishikawa

**DOI:** 10.1016/j.patter.2023.100794

**Published:** 2023-07-14

**Authors:** Daisuke Komura, Shumpei Ishikawa

**Affiliations:** 1 Department of Preventive Medicine, Graduate School of Medicine, The University of Tokyo, Tokyo, Japan

## Abstract

In this People of Data, Cell Press Community Review Scientific Editor Leia Judge talks to lead author Dr. Daisuke Komura and Principal Investigator Prof. Shumpei Ishikawa about their paper “Restaining-based annotation for cancer histology segmentation to overcome annotation-related limitations among pathologists,” which was published in the February issue of *Patterns*, and their experiences with Cell Press Community Review.

## Main text

### Can you provide a brief overview of the main findings and significance of your research?

**Figure:**
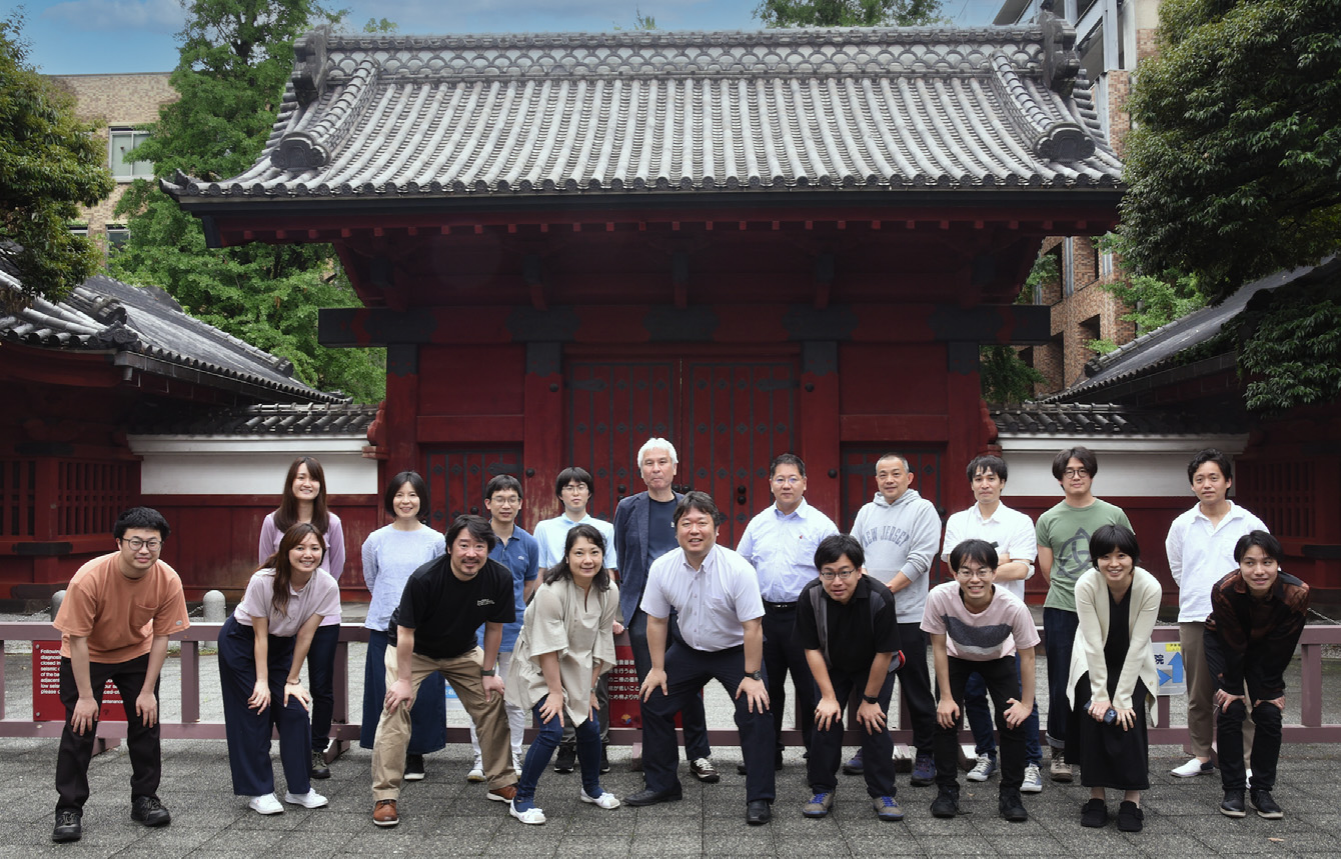
Lab members: (front row, fifth from the right) Dr. Daisuke Komura, (back row, fifth from right) Prof. Shumpei Ishikawa, with consent of the lab members photographed.

**Daisuke Komura & Shumpei Ishikawa:** We have developed a method for generating rich annotations for tissue and cell segmentation in digital pathology images of hematoxylin and eosin (H&E)-stained tumor tissue sections.^1^ Instead of the usual manual approach, we successfully generated many annotations at a level not possible by manual annotation using a process called immunofluorescence restaining. The importance of cell/tissue segmentation of H&E images lies not only in its role in pathological diagnosis, such as measuring tumor area, but also in understanding cancer pathology. Identifying the types of cells or tissues present and their spatial relationships to each other is critical to deciphering the pathogenesis of cancer. Importantly, our method’s use of antibodies also allows for the annotation of difficult-to-identify cells, which are often a challenge for human pathologists. We anticipate that this method will lead to the creation of more accurate segmentation models, improving both the accuracy and depth of cancer pathology analysis.

### What motivated you to pursue this particular research topic? Were there any specific challenges or gaps in the existing methods for cancer histology segmentation that you aimed to address?

**DK & SI:** Our lab has long been interested in using machine learning to aid pathological diagnosis and facilitate knowledge discovery from cancer pathology images. In our previous work published in *Cell Reports* in 2022,^2^ we treated histopathology images as textures and transformed them into feature vectors to find similar cases and correlate them with cancer genomic abnormalities. However, we felt that accurate identification of various cells such as lymphocytes and myeloid cells, in addition to tumor cells, was essential to understanding the context within these tissues.

As we began to examine existing datasets for cancer tissue segmentation, we encountered several challenges. First, our lab studies a variety of tumors, but existing datasets primarily cover tumors common in Western countries, such as colon and breast cancer. This limited the scope of our diverse research interests. Second, freeing up pathologists' time for annotation was impractical due to their diagnostic workload. Given these gaps, we felt a compelling need to develop a method that could efficiently generate many annotations across different tissues with minimal pathologist intervention, hence the motivation for this research.

### How did you decide to submit to Community Review? Were you familiar with Community Review beforehand?

**DK:** I have been aware of Community Review since its inception, have used it several times, and have found it to be a convenient system. We do a lot of genomic research and are familiar with the Cell Press journals, having even published a paper on pathology image analysis in *Cell Reports*.^2^ Cell Press also publishes papers on deep learning techniques for cell recognition, so I thought it would be a good platform for our research. However, it was unclear which specific journal would be the best fit for our study. In addition, the traditional submission process—submitting to one journal, facing possible rejection, and then resubmitting to another—can be time consuming and delay the dissemination of our research. Therefore, I decided to submit our work to Community Review to take advantage of its benefits, such as broader exposure to multiple journal editors and a faster, more transparent review process.

### Your manuscript underwent a unique publishing journey, starting with submission to several Cell Press journals and ultimately finding its home at *Patterns* via Community Review. Can you share your experience with this process, from submission to acceptance?

**DK & SI:** Indeed, our manuscript took an unconventional path before finding its rightful place in *Patterns*. First, we submitted our work to several Cell Press journals through Community Review, including *Cell*, *Cell Reports*, *Cell Systems*, and *Cancer Cell*. Although we did not receive interest from the editors of these journals, we did receive positive responses from the editors of *Patterns*, *Cell Reports Methods*, and *iScience*. Given their interest, we decided to proceed with the review process. After the review phase, we responded to the reviewers' comments and submitted our revisions. Both *Patterns* and *iScience* expressed interest in our work after revision. After considering the mission and scope of both journals, we ultimately chose *Patterns* for final submission. This experience highlighted the flexibility and openness of the community review process in helping us find the most appropriate platform for our research.

### *Patterns* was not among the journals you initially selected at submission; what are your thoughts on the value of Community Review and its ability to provide alternative pathways to publication that may not have been initially considered by authors?

**DK & SI:** While *Patterns* wasn’t initially on our list of potential journals, the Community Review process revealed its compatibility with our research. *Patterns* is a relatively new journal, and we weren’t entirely familiar with it. However, after it was suggested through the Community Review process, we realized that its concept and the papers it publishes are closely aligned with our work. The value of our research lies in the data we’ve generated and the process we’ve used to create it, which fits perfectly with *Patterns*'s data descriptor category. Without the recommendation of the Community Review, we may not have made this connection.

In recent years, the number of journals has increased, making it more difficult to understand the concepts of all of them and whether our paper would fit. In such an environment, the ability of the Community Review system to recommend appropriate outlets, even those not initially considered, is very useful. It accelerates the process of getting our research to more appropriate readers, increasing its overall impact.

### Restaining-based annotation is an innovative approach discussed in your paper. Could you elaborate on how this method works and why it is particularly useful for overcoming annotation-related limitations among pathologists?

**DK & SI:** Restaining-based annotation is a method in which we perform an initial H&E stain, destain the specimen, and then perform immunostaining with cell-type-specific antibodies. This process precisely aligns the positions of the H&E staining with the target cell types. This approach has two major advantages.

First, it can generate a tremendous amount of annotation compared to manual annotation by pathologists. This is particularly beneficial in places like Japan, where pathologists are incredibly busy—a technique that minimizes their involvement is very beneficial. Second, it can identify cells that even pathologists may have difficulty characterizing. While pathologists are experts in pathological diagnosis, they are not specifically trained in cell-type identification. They can estimate the relative proportions of different cell types in a field of view, but identifying individual cells, especially those with less distinct features such as myeloid cells or cells with atypical morphologies due to some reason such as distortion, is a challenging task even for them. Our restaining-based annotation method allows accurate annotation even for these cell types.

### The field of cancer histology segmentation is continuously evolving. How do you envision your research impacting this field? Have there been any new projects or collaborations that have emerged based on the datasets and code you published with your paper?

**DK & SI:** With our research, we expect to enable the segmentation of different tissues and cells in various cancer types on an unprecedented scale. This will not only advance research on segmentation methods but also stimulate new studies using the results of cancer tissue segmentation. For example, detailed analysis of the spatial relationships between tumor cells and various immune cells in the tumor microenvironment may lead to research on new prognostic factors or prediction of the efficacy of immunotherapy.

We’ve already received inquiries and requests for collaboration related to this study from several companies. In addition, since our lab’s goal is to support the pathology diagnostic process and extract knowledge from pathological tissue images, we’re pursuing several research projects that use the segmentation model trained on this dataset as a pre-processing step. Thus, our work has the potential to contribute significantly to the ongoing evolution of cancer histology analysis.

### Collaborations and interdisciplinary approaches are often vital in scientific research. Did your work involve collaborations with experts from other domains, such as pathologists, computer scientists, or other bioinformaticians? If so, how did these collaborations contribute to the success of your study?

**DK & SI:** Our study required close collaboration between computer scientists and pathologists or pathology researchers. Tasks such as selection of appropriate cases, selection of antibodies, verification of staining conditions, and evaluation of generated annotations could only be performed by pathology researchers, while data analysis was performed by computer scientists. After data acquisition, the pathology researchers would revise case selection and antibody selection based on the computer scientists' analysis results, demonstrating the indispensability of this synergy. A one-way interaction would not have produced the high-quality data we did.

Fortunately, our lab is unique in having both computer scientists and pathologists. Having everyone in the same room allows for a smooth exchange of ideas. As a result, we have been able to manage all processes from case selection, antibody selection, staining, scanning, and analysis within the lab, streamlining our research.

**DK:** In addition, I am uniquely positioned for this endeavor. After earning a PhD in engineering with a focus on bioinformatics, I entered medical school. My background in pathology made it easier to work with pathologists. This interdisciplinary approach undoubtedly contributed to the success of our study.

### What are your thoughts on the future of peer review and scientific publishing? Do you see any emerging trends or changes that may shape the landscape in the coming years?

**DK:** As the number of researchers and journals continues to grow, we are facing a bottleneck in the peer review process. The shortage of reviewers is becoming more of an issue, and I too am overwhelmed with review requests. The current system often results in a manuscript being reviewed from scratch by different reviewers each time it’s submitted to a new journal. Given this scenario, we need to look at ways to make the review process more efficient.

In addition, research seems to be accelerating, especially in areas such as machine learning, with generative AI being a prime example. The traditional process of publishing papers after a review cycle often cannot keep up with this pace. Therefore, for some disciplines, a system where papers are published in venues such as preprint servers and then peer reviewed may prove effective. However, maintaining the quality of research and ensuring appropriate revisions based on valuable reviewer feedback remains critical. Therefore, I believe that we need a new system that balances these two aspects, addressing the need for speed while preserving the quality of scientific work.

### Lastly, what advice would you give to researchers who may be considering submitting their work through the Community Review process or exploring alternative publication pathways? Based on your own experience, what key factors should they consider when deciding on a journal for their research?

**DK:** If you are not sure which journal is the best fit for your work, or if you have several potential journals, the Community Review process could streamline your publication process. The submission process can be time consuming, and the Community Review system may be a worthwhile consideration to free up valuable research time. The value of a paper is determined not only by its publication but also by how it becomes a cornerstone for other studies after publication. Therefore, when choosing a journal, it’s important to consider whether your work will reach a broad audience in your field. My advice is to choose a journal that ensures that your work will be seen and have an impact among relevant researchers, rather than focusing solely on the prestige of the journal.

### About Cell Press Community Review

Cell Press Community Review provides a collaborative approach to peer review and publication within Cell Press, offering authors alternative pathways to share their work. This streamlined process saves time and identifies the best-fit journal for the research, with >80% of papers being offered peer review at one or more of the participating Community Review journals.
